# Prevalence of post-intensive care syndrome in mechanically ventilated patients with COVID-19

**DOI:** 10.1038/s41598-022-11929-8

**Published:** 2022-05-13

**Authors:** Kapil Nanwani-Nanwani, Lorenzo López-Pérez, Carola Giménez-Esparza, Inés Ruiz-Barranco, Elena Carrillo, María Soledad Arellano, Domingo Díaz-Díaz, Beatriz Hurtado, Andoni García-Muñoz, María Ángeles Relucio, Manuel Quintana-Díaz, María Rosario Úrbez, Andrés Saravia, María Victoria Bonan, Francisco García-Río, María Luisa Testillano, Jesús Villar, Abelardo García de Lorenzo, José Manuel Añón

**Affiliations:** 1 Intensive Care Unit, Hospital Universitario La Paz, Paseo de la Castellana 261, 28046 Madrid, Spain; 2 Intensive Care Unit, Hospital Universitario Infanta Leonor, Madrid, Spain; 3 Intensive Care Unit, Hospital Vega Baja, Orihuela, Alicante, Spain; 4 Instituto de Investigación Sanitaria del Hospital Universitario La Paz (IdiPAZ), Madrid, Spain; 5 Department of Physical Medicine and Rehabilitation, Hospital Universitario La Paz, Madrid, Spain; 6 Department of Psychiatry, Hospital Universitario La Paz, Madrid, Spain; 7 Department of Respiratory Medicine, Hospital Universitario La Paz, Madrid, Spain; 8 Centro de Investigación Biomédica en Red de Enfermedades Respiratorias (CIBERES), Instituto de Salud Carlos III, Madrid, Spain; 9 Department of Pharmacy, Hospital Universitario La Paz, Madrid, Spain; 10 Research Unit, Hospital Universitario Dr. Negrín, Las Palmas de Gran Canaria, Spain; 11 Li Ka Shing Knowledge Institute, St. Michael’s Hospital, Toronto, ON Canada; 12 Universidad Autónoma de Madrid, Madrid, Spain

**Keywords:** Outcomes research, Respiratory distress syndrome

## Abstract

Coronavirus disease 19 (COVID-19) patients usually require long periods of mechanical ventilation and sedation, which added to steroid therapy, favours a predisposition to the development of delirium and subsequent mental health disorders, as well as physical and respiratory sequelae. The aim of this study was to determine the prevalence of post-intensive care syndrome (PICS) at 3 months after hospital discharge, in a cohort of mechanically ventilated patients with severe acute respiratory syndrome coronavirus 2 (SARS-CoV-2). An ambispective, observational study was conducted in three hospitals with intensive care unit (ICU) follow-up clinics. We studied adults who survived a critical illness due to SARS-CoV-2 infection requiring invasive mechanical ventilation. A physical (muscle strength and pulmonary function), functional [12-Item Short Form Health Survey (SF-12), and Barthel score], psychological [hospital anxiety and depression (HADS) and posttraumatic stress disorder symptom severity scales], and cognitive [Montreal cognitive assessment (MoCA) test] assessment were performed. A total of 186 patients were evaluated at 88 days (IQR 68–121) after hospital discharge. Mean age was 59 ± 12 years old, 126 (68%) patients were men, and median length of mechanical ventilation was 14 days (IQR 8–31). About 3 out of 4 patients (n = 139, 75%) met PICS criteria. Symptoms of cognitive and psychiatric disorders were found in 59 (32%) and 58 (31%) patients, respectively. Ninety-one (49%) patients had muscle weakness. Pulmonary function tests in patients with no respiratory comorbidities showed a normal pattern in 93 (50%) patients, and a restrictive disorder in 62 (33%) patients. Also, 69 patients (37%) were on sick leave, while 32 (17%) had resumed work at the time of assessment. In conclusion, survivors of critical illness due to SARS-CoV-2 infection requiring mechanical ventilation have a high prevalence of PICS. Physical domain is the most frequently damaged, followed by cognitive and psychiatric disorders. ICU follow-up clinics enable the assistance of this vulnerable population.

## Introduction

The post-intensive care syndrome (PICS) represents a constellation of cognitive, psychiatric, physical, and pulmonary disorders frequently seen following admission into intensive care units (ICUs) in pre-pandemic studies^1^. At ICU discharge, nearly all survivors of critical illness experience impairments in one or more PICS domains. At 3 and 12 months, 64% and 56% of survivors experience one or more new post-intensive care problems, respectively, and co-occurrence is common^2,3^. Risk factors for developing PICS include longer periods of mechanical ventilation, delirium, treatment with steroids, vasoactive drugs and sedation, among others^1^.

Coronavirus disease 19 (COVID-19) has caused a worldwide surge in critical care demand. Up to 20% of hospitalized patients infected with the severe acute respiratory syndrome coronavirus 2 (SARS-CoV-2) require admission into the ICU, out of which more than 88% require endotracheal intubation and invasive mechanical ventilation (MV)^4^. Moreover, COVID-19 patients usually require longer periods of MV and sedation than non-COVID-19 critically ill patients, which added to steroid therapy, favour a predisposition to the development of delirium and subsequent mental health disorders, as well as physical and respiratory sequelae^5^.

Short and long-term sequelae of SARS-CoV-2 infection in patients admitted in ICU have been described^6–22^. However, PICS (in all 3 domains) in post-critical care mechanically ventilated COVID-19 patients has not been extensively studied^10,11,17,19^.

The aim of this study was to determine the prevalence of PICS in a cohort of mechanically ventilated SARS-CoV-2 patients, assessed after 3 months of hospital discharge, in the ICU follow-up consultation facilities of three major hospitals in Spain.

## Methods

### Study design

The study was designed in accordance with the Declaration of Helsinki^23^. We conducted an ambispective, observational study in three hospitals with ICU follow-up consultation facilities. We studied adult patients (≥ 18 years old) admitted to the ICU due to severe SARS-CoV-2 infection, requiring invasive MV and who were alive at the time of hospital discharge. Patients with previous severe psychiatric conditions, cognitive deficits, and any sort of functional dependency were excluded. Exclusion criteria also included patients from a different geographical area who were not willing to come for assessment to our centres and patients who refused to sign the informed consent form.

Data regarding demographic variables, treatment drugs (hypnotics, analgesics, muscle relaxants, corticoids and vasopressors), delirium, management procedures [length of MV, renal replacement therapy (RRT), extracorporeal membrane oxygenation (ECMO), extracorporeal CO_2_ removal (ECCO_2_R), tracheostomy] and complications [such as nosocomial infections, venous thromboembolism (VTE), pulmonary embolism (PE), and stroke] during the ICU stay were retrospectively registered from the patient´s electronic medical record. Only hyperactive delirium was registered in our analysis. Prolonged MV (PMV) was defined as the need for invasive MV for more than 14 days.

### Follow-up assessment

In line with our usual clinical practice^24^, a follow-up appointment was arranged for all patients at three months after hospital discharge. This consultation included anamnesis and an assessment of potentially affected domains where PICS was evaluated: mental status, cognition, muscle strength, pulmonary function, dependence and functional status. All the variables related to this assessment were prospectively recorded.

Mental status was evaluated using the Hospital Anxiety and Depression Scale (HADS)^25^ and the Post-Traumatic Stress Disorder (PTSD) symptom severity scale^26^. The HADS scale combines two 7-item subscales evaluating symptoms of depression (HADS-D subscale) and anxiety (HADS-A subscale). We used a score of ≥ 8 in the anxiety or depression subscale to identify clinically relevant anxiety or depression. The PTSD symptom severity scale is a 0 to 3 scoring scale according to the frequency and intensity of symptoms, it has been validated in the Spanish population^26^ and has 17 items: five refer to re-experiencing symptoms (range 0 to 15 points), seven to avoidance symptoms (range 0 to 21 points), and five to symptoms of increased activation (range 0 to 15 points). A symptom is considered when it is scored with at least 2 points. In order to consider PTSD, the presence of one symptom is required in section A (re-experiencing), three symptoms in section B (avoidance), and two symptoms in section C (increased activation).

Cognition was assessed using the Montreal Cognitive Assessment (MoCA) test^27^. The MoCA test evaluates global cognitive function, including executive function, attention/working memory, episodic memory, and language. Total score ranges from zero to 30. Mild cognitive impairment was defined as a score of 18–25, moderate as a score of 10–17, and severe when the score is less than 10^2^.

Muscle strength was assessed using a handgrip dynamometry, a basic method that is standardized by age groups and sex^28^. We used an electronic digital LCD device (Camry^®^, General ASDE SA, Spain, 93/42/CEE). Reference values are based on the study by Luna et al.^29^ in which they consider cut-off points of 85% from those obtained in a Spanish population of healthy volunteers (267 women and 229 men) aged between 17 and 97 years.

Pulmonary function was assessed by spirometry (Sibelmed^®^ datospir touch. SIBEL. S.A.U. Barcelona, Spain). Protocol and interpretation were based on the 2005 American Thoracic Society (ATS) and European Respiratory Society (ERS) statements^30^, and Quanjer equations were used as reference values^31^.

Dependence was assessed using the Barthel score^32^. The Barthel score has 10 subheadings related to activities of daily living (ADL). Scoring ranges from zero to 100. A score of 100 is defined as being capable of ADL as well as complete self-care.

Quality of life was assessed using the 12 Item Short Form Healty Survey (SF-12)^33^, wich is a health-related quality of life questionnaire consisting of twelve items that measure eight health domains associated with physical and mental health. Physical health-related domains include general health, physical activities, usual role activities and body pain. Mental health-related scales include vitality, social activities, emotion influenced limitations in role activities and general mental health. The instrument was self-administered and two summary scores of the SF-12—physical and mental health—were calculated using the weighted means of the eight domains. A score under 50 indicates a poor health-related quality of life in relation to the reference population, whereas a score above 50 indicates good health-related quality of life.

Patients met PICS criteria if they had derangements in at least one of the domains assessed with the scales and tools used to detect long-term cognition, mental health, and physical function, as previously detailed, according to Needham et al.^1^ and adapted from Mikkelsen et al.^2^. A summary of outcome measures, including domains assessed, scale details, and interpretation, are shown in Table 1.

**Table 1:** Outcomes measured to establish PICS criteria.

Scale/assessment tool used	PICS domain	Cut-off
Spirometry	Physical/pulmonary	Impairment in spirometry pattern according to ATS/ERS statements^30,31^
Dynamometry	Physical/neuromuscular	< 85% of healthy volunteers according to Luna et al.^29^
Barthel score	Physical/dependence	Score < 95^32^
HADS-A	Psychiatric	Score ≥8 ^25^
HADS-D	Psychiatric	Score≥8^25^
PTSD symptom severity scale	Psychiatric	One symptom, three symptoms and two symptoms in sections A (re-experiencing), B (avoidance) and C respectively (increased activation)^26^
MoCA test	Cognitive	Score: 18–25 Mild cognitive impairment^2^
		Score: 10–17: Moderate cognitive impairment^2^
		Score < 10: Severe cognitive impairment^2^

*PICS* post-intensive care syndrome, *HADS-A* Hospital Anxiety and Depression Scale-Anxiety, *HADS-D* Hospital Anxiety and Depression Scale-Depression, *PTSD* Post-traumatic stress disorder, *MoCA* Montreal cognitive assessment.

### Statistical methods

Quantitative variables were expressed as mean ± standard deviation, or median and interquartile range (IQR) for variables not fitting a normal distribution according to Kolmogornov–Smirnov’s test. Chi-square test was used for analysing the association between qualitative variables. For qualitative variables in which the “n” was < 20 or any theoretical value was < 5, Fisher’s exact test was used. Differences were considered statistically significant if the *p*-value was < 0.05.

Relationship between the quantitative variables used as PICS criteria and mental or physical SF-12 component was assessed by Pearson correlation analysis. For assessing the independent association of each variable with PICS, a multivariate logistic regression analysis including those variables with a p < 0.05 in bivariate analysis was developed. The method used for regression was forward elimination.

Data were processed by the Statistical Package for the Social Sciences Software (version 26.0) for Windows (SPSS Inc, Chicago, IL).

### Ethics approval

The study was approved by the Ethical Committee of Clinical Research of University Hospital La Paz, IdiPAZ, Madrid, Spain (reference number: #PI-4325) and was adopted for all participating centres, as required by Spanish legislation. All patients provided written informed consent for its inclusion in the study.

## Results

**Figure 1:**
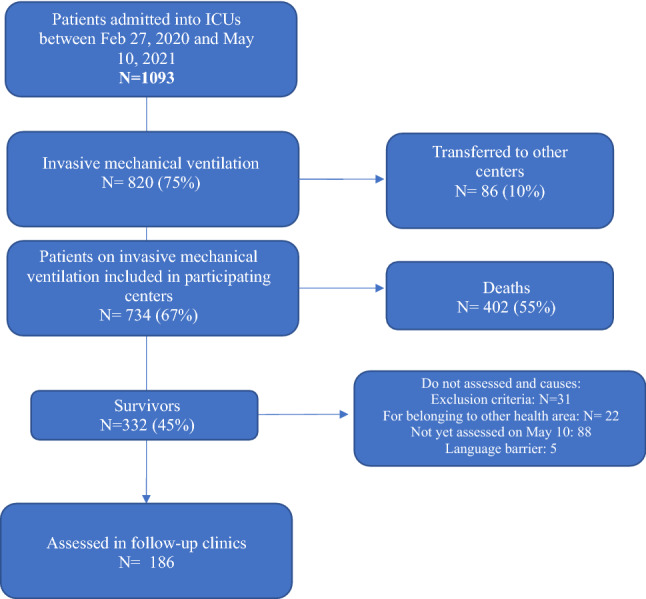
Flowchart showing the patients included into the study.

From February 27th, 2020 to May 10th, 2021, a total of 1093 patients with acute respiratory failure due to SARS-CoV-2 were admitted to the ICUs of participating hospitals: 820 patients (75%) required invasive MV and 86 (10%) were transferred to other centres for lack of ICU beds. Out of the remaining 734 patients, 332 (45%) survived to hospital discharge. On May 10th, those patients (n = 88) who met inclusion criteria but did not complete the period from hospital discharge, and had not been assessed in follow-up clinic had to be excluded. Other 58 patients were excluded due to other reasons (had exclusion criteria, were from other health areas, or due to language barriers). Finally, 186 patients were enrolled in the present study (Fig. 1). Patients were assessed in the ICU follow-up consultation at 88 days (IQR 68–121) after hospital discharge [a median of 110 days (IQR 84–167) after ICU discharge]. Demographic and clinical features are shown in Table 2.

**Table 2:** Demographic and clinical data during ICU admission.

	N = 186
Sex, male n (%)	126 (68)
Age, mean (SD), years	59 (12)
BMI prior to ICU admission, mean (SD)	31 (5)
**Ethnicity**	
Latin American, n (%)	55 (30)
Others, n (%)	131 (70)
**Comorbidities**	
Arterial hypertension, n (%)	93 (50)
Dyslipidemia, n (%)	65 (35)
Diabetes, n (%)	32 (17)
Cancer, n (%)	14 (7)
Hypothyroidism, n (%)	13 (7)
Alcohol abuse, n (%)	11 (6)
COPD, n (%)	9 (5)
Chronic liver disease, n (%)	8 (4)
Current smoker, n (%)	7 (4)
Solid organ transplant, n (%)	7 (4)
Chronic renal failure, n (%)	5 (3)
Ischemic heart diseases, n (%)	5 (3)
**Clinical data**	
APACHE II, mean (SD)	13 (5)
Length of stay in ICU, median (IQR), days	27 (14–56)
Length of stay in hospital, median (IQR), days	54 (29–81)
Length of mechanical ventilation, median (IQR), days	14 (8–31)
Hypnosis with Midazolam n (%)	118 (63)
Days, mean (SD)	9 (8)
Hypnosis with Propofol n (%)	184 (99)
Days, mean (SD)	14 (11)
Hypnosis with Midazolam and Propofol n (%)	115 (63)
Hypnosis with Ketamine n (%)	28 (15)
Days, mean (SD)	7 (5)
Analgesia with Fentanyl n (%)	160 (86)
Days, mean (SD)	17 (16)
Analgesia with Remifentanil n (%)	47 (25)
Days, mean (SD)	10 (9)
Patients who needed paralysis, n (%)	157 (84)
Days, mean (SD)	7 (5)
Patients who needed vasopressors n (%)	155 (83)
Re-endotracheal intubation, n (%)	13 (7)
Patients who needed tracheostomy, n (%)	87 (47)
Patients who needed RRT, n (%)	15 (8)
Patients who needed support with ECMO, n (%)	5 (3)
Patients who needed support with ECCO_2_R, n (%)	3 (2)
Patients treated with steroids, n (%)	163 (88)
Dexamethasone, n (%)	157 (84)
Methylprednisolone, n (%)	6 (3)
Hyperactive delirium, n (%)	119 (64)
Nosocomial infection during ICU stay, n (%)	114 (61)
Patients with pulmonary embolism, n (%)	44 (24)
Patients with deep vein thrombosis, n (%)	9 (5)
Patients with ischaemic stroke, n (%)	4 (2)
Hospital discharge with home oxygen, n (%)	41 (22)

*BMI* body mass index, *COPD* chronic obstructive pulmonary diseases, *APACHE II* acute physiology and chronic health evaluation II, *SD* standard deviation, *ICU* intensive care unit, *IQR* interquartile range, *RRT* renal replacement therapy, *ECMO* extracorporeal membrane oxygenation, *ECCO*_*2*_*R* extracorporeal CO_2_ removal.

Thirty-three patients (18%) were transferred to a rehabilitation centre after being discharged from the hospital, while 41 patients (22%) needed domiciliary oxygen, and 52 patients (28%) needed home care.

At the time of assessment at the ICU follow-up clinic, 139 patients (75%) met PICS criteria: 86 (46%), 40 (21%) and 13 (7%) patients had derangements of one, two or three PICS domains, respectively.

Regarding physical assessment, the most common symptoms were dyspnea (n = 106, 57%), muscle weakness (n = 91, 49%), and joint pain (n = 83, 45%) (Table 3). After excluding 7 patients with a history of chronic respiratory disease, 93 patients (50%) had a normal pulmonary function pattern, in 16 patients (10%) spirometry tests were not evaluable due to lack of patient’s collaboration, 62 patients (33%) showed a restrictive disorder, 5 patients (3%) had a mixed (obstructive-restrictive) disorder, and 3 patients (2%) showed an obstructive spirometry pattern. Values of spirometry are shown in Table 3.

**Table 3:** Clinical results obtained in the outpatient assessment.

	N = 186
Dyspnea, (mMRC), n (%)	
0	80 (43)
1	57 (31)
2	29 (15)
3	17 (9)
4	3 (2)
Muscular weakness, n (%)	91 (49)
Joint pain, n (%)	83 (45)
Joint limitation, n (%)	53 (28)
Paraesthesia, n (%)	45 (24)
Alopecia, n (%)	42 (23)
Anosmia, n (%)	21 (11)
Headache, n (%)	18 (10)
Dysphagia, n (%)	5 (3)
**SF-12 score**	
- *Physical*	
--Physical function, mean (SD)	41 (12)
--Role physical, mean (SD)	44 (12)
--Bodily pain, mean (SD)	40 (13)
--General health, mean (SD)	38 (11)
- *Mental*	
--Vitality, mean (SD)	48 (12)
--Social functioning, mean (SD)	47 (12)
--Role emotional, mean (SD)	42 (13)
--Mental health, mean (SD)	49 (10)
- *Summary*	
--Physical component summary score, mean (SD)	38 (13)
--Mental component summary score, mean (SD)	49 (12)
Barthel index, mean (SD)	95 (11)
MoCA test, mean (SD)	25 (4)
HADS-A mean (SD)	6 (5)
HADS-D mean (SD)	5 (4)
FEV_1_ mean, % pred. (SD)	88 (18)*
FVC mean, % pred. (SD)	83 (16)*
FEV_1_/FVC, %, mean (SD)	84 (9)*

*mMRC* Modified Medical Research Council (mMRC) dyspnea scale, *SF-12* short form-12 health survey, *MoCA* Montreal Cognitive Assessment (MoCA) test, *SD* standard deviation, *HADS-A* Hospital Anxiety and Depression Scale-Anxiety, *HADS-D* Hospital Anxiety and Depression Scale-Depression, *FEV*_*1*_ forced expiratory volume at 1 s, *FVC* forced vital capacity.

*Seven patients with a history of chronic respiratory disease, were excluded.

Cognitive and psychiatric disorders were found in 59 (32%), and 58 (31%) patients respectively. In 47 patients (25%), cognitive disorders were mild, whereas 12 patients (6%) had moderate cognitive disorders. In our study, no patients showed severe cognitive disorders. Symptoms of psychiatric disorders are shown in Table 4.

**Table 4:** Psychiatric symptoms.

Symptoms	186 Patients n (%)
Depression, anxiety and postraumatic stress disorder	26 (14)
Depression and anxiety	6 (3)
Anxiety	6 (3)
Depression	4 (2)
Postraumatic stress disorder	8 (4)
Postraumatic stress disorder and anxiety	5 (3)
Postraumatic stress disorder and depression	3 (2)

Summary of MoCA test, Barthel score, and SF-12 score are shown in Table 3. The physical component of the SF-12 showed a correlation with the degree of dyspnea (mMRC) (r =  − 0.32, p < 0.001) and Barthel scale (r = 0.49 p < 0.001), whereas the SF-12 mental component was strongly related to HADS-A (r =  − 0.68, p < 0.001) and HADS-D scales (r =  − 0.67, p < 0.001).

PICS was associated with PMV (p = 0.01), use of benzodiazepines (p = 0.002), and nosocomial infection (p = 0.04) (Table 5). Associations between these variables and domains of PICS are shown in Table 6. Nosocomial infection, use of benzodiazepines, and PMV were included in the stepwise multiple regression logistic analysis which only retained as independent variable the PMV (adjusted odds ratio: 2.271, 95%CI 1.140–4.524, p = 0.020).

**Table 5:** Association between complications/procedures during ICU admission and PICS (N = 186).

Procedures	PICS	No PICS	p
MV < 7 days, n (%)	23 (64)	13 (36)	0.09
MV: 7–14 days, n (%)	41 (69)	18 (31)	0.2
MV > 14 days, n (%)	75 (82)	16 (18)	0.01
Midazolam, n (%)	97 (82)	21 (18)	0.002
Propofol, n (%)	137 (74)	47 (26)	0.5
Ketamine, n (%)	24 (86)	4 (14)	0.1
Fentanyl, n (%)	122 (76)	39 (24)	0.2
Remifentanil, n (%)	32 (68)	15 (32)	0.2
Paralysis, n (%)	118 (75)	39 (25)	0.7
Steroids, n (%)	121 (74)	42 (26)	0.3
Vasopressors, n (%)	119 (77)	36 (23)	0.1
Nosocomial infection, n (%)	91 (80)	23 (20)	0.04
RRT, n (%)	13 (87)	2 (13)	0.3
ECMO, n (%)	5 (100)	0 (0)	0.1
ECCO_2_R, n (%)	3 (100)	0 (0)	0.1
Delirium, n (%)	94 (79)	25 (21)	0.07

*ICU* intensive care unit, *PICS* post-intensive care syndrome, *MV* mechanical ventilation, *RRT* renal replacement therapy, *ECMO* extracorporeal membrane oxygenation, *ECCO*_*2*_*R* extracorporeal CO_2_ removal.

**Table 6:** Association of the variables related to PICS with psychiatric, cognitive and physical domains (N = 186).

	MV > 14 days	MV ≤ 14 days	p
PICS, n (%)	75 (54)	64 (46)	0.01
Cognitive derangement, n (%)	34 (58)	25 (42)	0.2
Depression, n (%)	22 (56)	17 (44)	0.2
Anxiety, n (%)	23 (54)	20 (46)	0.4
PTSD, n (%)	23 (55)	19 (45)	0.3
Muscle weakness, n (%)	55 (66)	28 (34)	0.0001
Abnormal spirometry, n (%)	40 (55)	33 (45)	0.1

	Benzodiazepines	No benzodiazepines	
PICS, n (%)	97 (70)	42 (30)	0.002
Cognitive derangement, n (%)	38 (64)	21 (36)	0.8
Depression, n (%)	27 (69)	12 (31)	0.3
Anxiety, n (%)	33 (77)	10 (23)	0.03
PTSD, n (%)	30 (71)	12 (29)	0.2
Muscle weakness, n (%)	59 (71)	24 (29)	0.05
Abnormal spirometry, n (%)	52 (71)	21 (29)	0.1

	Nosocomial infection	No nosocomial infection	
PICS, n (%)	91 (65)	48 (35)	0.04
Cognitive derangement, n (%)	39 (66)	20 (34)	0.3
Depression, n (%)	24 (62)	15 (38)	0.9
Anxiety, n (%)	24 (56)	19 (44)	0.4
PTSD, n (%)	24 (57)	18 (43)	0.5
Muscle weakness, n (%)	62 (75)	21 (25)	0.001
Abnormal spirometry, n (%)	50 (69)	23 (31)	0.06

*PICS* post-intensive care syndrome, *MV* mechanical ventilation, *PTSD* post-traumatic stress disorder.

Eighty-six patients (46%) were transferred to other clinical specialists, after assessing their needs. Thirty-one patients (17%) were transferred to the Department of Mental Health, 19 patients (10%) were transferred to Physical Medicine and Rehabilitation Department, and 7 patients (4%) were transferred to both departments. Thirty-four patients (18%) were already treated by those departments and did not required further transfer. Twenty-nine patients (16%) were transferred to other specialists (including respiratory medicine, internal medicine, and neurology, among others).

Regarding the social aspects of life, 32 patients (17%) had resumed work at the time of assessment, 69 patients (37%) were on sick leave, 57 patients (31%) were retired prior to hospital admission, 7 patients (13%) remained unemployed, and 15 patients (8%) were housekeepers (as prior to hospital admission). Eighty patients (43%) had resumed driving, and 48 patients (26%) had normalized their sexual activities.

## Discussion

The main finding of this study is that about three out of four survivors of severe COVID-19 met PICS criteria: 46%, 21%, and 7% of them had derangements of one, two, or three PICS domains, respectively. Physical domain was the most frequently damaged.

Prior to our work, post-ICU COVID-19 related sequelae has been assessed in other reports.

Some of them with larger samples, but with a smaller number of patients requiring invasive mechanical ventilation^6,7,21^. Only two studies^17,19^ have assessed the three PICS domains focused on ventilated patients: the first one^17^ assessed a cohort of 47 patients 6 months after hospital discharge, and the second one^19^ analyzed a cohort of 178 patients at 3 and 12 months after hospital discharge.

We found a prevalence of PICS of 75% with a 28% co-occurrence of symptoms, with dyspnea being the most frequent symptom (57%). The highest prevalence of PICS in COVID-19 patients reported to date has been 91% in 45 patients (out of which 90% had been on MV) and 58% had at least two main domains affected^16^. Of note, follow-up consultations in that study^16^ were via telematics at 1 month after hospital discharge. Gamberini et al.^19^ found that most of the patients reported persistent symptoms 1 year after ICU discharge, with dyspnea (58%) being the most frequent symptom. Heesakers et al.^21^, also 1 year after ICU treatment, reported physical symptoms in 74% (weakness 38.9%), mental symptoms in 26% and cognitive symptoms in 16% of the patients, of whom 81% underwent mechanical ventilation. Overall, 31% of the survivors reported symptoms in at least 2 domains, and 10% experienced symptoms in all 3 domains.

Main conditions associated with PICS included large duration of MV, treatment with benzodiazepines, and nosocomial infection, although PMV was the independent variable associated to PICS. PMV and deep sedation have been classically associated with PICS^1^. In the COVID-19 era, a large number of patients required PMV and deep sedation (with the coexistence of two or more hypnotics) and relaxation to facilitate MV in the prone position. There have been substantial concerns about respiratory sequelae due to COVID-19. At 3 months after hospital discharge, we observed that pulmonary function was normal in over 50% of patients in our study. Also, 43% did not manifest dyspnea, and in those who did, the mean modified Medical Research Council (mMRC) dyspnea scale^34^ was 1.7 ± 0.9 which corresponds to a low symptom intensity (mainly breathlessness only on strenuous exercise). Other authors^35^ have published similar spirometry results. Morin et al.^7^ included functional and morphological assessment, reporting that severe pulmonary sequelae were infrequent, although all had experienced a severe or very severe form of COVID-19 associated pneumonia. In contrast, both, prevalence of restrictive spirometry results in our patients, was slightly higher compared to COVID-19 pneumonia patients who did not require ICU admission^36^. Mean SF-12 physical and mental summary scores in our cohort was lower when compared to previous reports at three months of follow-up^20^. These findings can be explained by a longer ICU stay and PMV. However, in line with the results of these authors^20^ we also found a correlation between degree of dyspnea and physical component of SF-12.

The published prevalence for cognitive and psychiatric disorders in post-critical COVID-19 patients covers a wide range, probably due to methodological heterogeneity among studies. Psychiatric disorders have been described in up to 49%^16^ while cognitive disturbances reach 57%^35^ at 1 month and 6 weeks of follow-up respectively. At 1 year following ICU treatment, mental symptoms were reported by 38%^19^ and cognitive symptoms by 16%^21^.

Unlike other pre-pandemic^37,38^ and pandemic^8,16,19^studies, our work has been carried out using face-to-face consultation. The scales we have used are recommended for these entities^2^ except for PTSD assessment. Due to easy-to-use characteristics, PTSD was evaluated using the symptom severity scale^26^, a structured interview developed in Spain that takes into account severity and intensity of symptoms. The main drawback of this scale is that it uses DSM-IV criteria as a reference.

The prevalence of delirium in ICU patients is estimated between 32 and 87%, although these figures vary considerably depending on whether the studied population had received MV^39^. All patients in our study underwent MV, and due to excessive workload during the pandemic, delirium and PICS prevention measures were inapplicable^40,41^. Prevalence of hyperactive delirium was found to be lower than previous published experiences, in COVID-19^42^, but somehow higher than the figure published in a large study of COVID-19 population^41^. No association between benzodiazepines and worse long-term cognitive scores was found, as described in previous studies^43^. Despite a 64% prevalence of hyperactive delirium, no influence on subsequent cognitive assessment was established, unlike other studies specifically designed for this purpose with a larger sample size^43,44^. Of note, since our study was not specifically designed for the prevalence of delirium, only hyperactive delirium was registered due to the diagnostic challenge posed by hypoactive delirium. Unlike prepandemic studies, hyperactive delirium is much more frequent than hypoactive delirium in COVID-19 patients^42^. Nevertheless, the hypothesis about a possible neuro-invasive potential of SARS-CoV-2^45^ and its influence on mental or psychiatric disorders remains open, since to date, its pathophysiology is poorly understood^39^.

Data on socio-occupational issues is absent in most studies. In our study, almost a third of previously active patients had resumed work within 88 days after hospital discharge, 43% had resumed driving, and about 25% had normalized their sexual life. Of note, two-thirds of our patients had a Barthel score of 100 points. These results differ drastically from those published by Rousseau et al.^46^ in 32 COVID-19 patients (where 30 patients required MV) who reported that only 6% of patients fully recovered and had normal MoCA, IES-R and Barthel scores three months after hospital discharge.

Our study has several strengths. First, our group has experience in post-ICU follow-up consultation since 2016^47^ and post-ICU follow-up is part of our usual clinical practice. Second, participating ICUs followed the same protocol, reducing heterogeneity among centres. Third, this is the largest study involving mechanically ventilated COVID-19 patients. Fourth, all patients in our study were assessed in a face-to-face consultation room with an intensivist and the support of a “post-ICU-team” (physiatrists, psychiatrists, psychologists and physiotherapists). No patient was assessed by phone or any other telematics mean. However, we acknowledge some limitations of our study. First, our study is an uncontrolled study design and, therefore, comparison with non-COVID-19 patients could not be established. Second, since our design is ambispective, some variables were retrospectively recorded. Third, 49% of studied patients were survivors of the first wave, the most devastating and challenging wave in which patient individualization and PICS prevention was inapplicable, along with changes in treatment protocol and patient upmake over time. Fourth; although according to our methodology patients with psychiatric impairment, severe cognitive impairment and patients with severe neuromuscular or neurological diseases were excluded from the PICS evaluation, baseline like HADS scale, MOCA test or muscle strength are unknown. Many of complaints related to these domains are often underdiagnosed. All of this probably may have overestimated its incidence in this patient population. Fifth, although our study is the largest cohort of mechanically ventilated COVID-19 patients, sample size is still limited to draw definitive conclusions. Participation of more centres would have been desirable to enlarge sample size. However, the number of centres with ICU follow-up clinics is still limited and some of them were forced to close due to the pandemic. Sixth, pulmonary function was assessed using forced spirometry. This test has obvious limitations, especially if it is not accompanied by other diagnostic procedures. Seventh, the possibility of performing lung morphological analysis at 3 months exceeded the objectives of the present study, which focused mainly on evaluating classically described PICS in a population of critical COVID-19 patients.

## Conclusions

This is the largest study addressing PICS in SARS-CoV-2 mechanically ventilated patients, assessed in follow-up ICU clinics. About three out of four survivors of severe COVID-19 meet PICS criteria. Physical domain is the most frequently damaged, followed by cognitive and psychiatric disorders. In line with the findings by other authors^48^, ICU follow-up clinics allow the assistance of this vulnerable population, as well as making advances in the understanding of PICS and COVID-19 sequelae.

## Data Availability

The datasets during and/or analyzed during the current study are available from the corresponding author on reasonable request.

## Abbreviations

ADL: Activities of daily living
ATS: American Thoracic Society
BMI: Body mass index
COPD: Chronic obstructive pulmonary diseases
COVID-19: Coronavirus disease-2019
ECCO_2_R: Extracorporeal CO_2_ removal
ECMO: Extracorporeal membrane oxygenation
ERS: European Respiratory Society
FEV_1_: Forced expiratory volume at 1 s
FVC: Forced vital capacity
HADS: Hospital Anxiety and Depression Scale
ICU: Intensive care unit
IQR: Interquartile range
mMRC: Medical Research Council Dyspnea Scale
MoCA: Montreal cognitive assessment
MV: Mechanical ventilation
PE: Pulmonary embolism
PICS: Post-intensive care syndrome
PMV: Prolonged mechanical ventilation
PTSD: Post-traumatic stress disorder
RRT: Renal replacement therapy
SF-12: 12-Item Short Form Health Survey
VTE: Venous thromboembolism
